# Pharmaceutical quality of seven brands of diclofenac tablet on the Saudi market

**DOI:** 10.1186/s13104-020-05385-8

**Published:** 2020-11-26

**Authors:** Muhammad M. Hammami, Reem AlSwayeh, Rajaa F. Hussein

**Affiliations:** 1 Clinical Studies and Empirical Ethics Department, King Faisal Specialist Hospital and Research Centre, P O Box # 3354 (MBC 03), Riyadh, 11211 Saudi Arabia; 2 Alfaisal University College of Medicine, Riyadh, Saudi Arabia

**Keywords:** Diclofenac potassium immediate-release, Diclofenac sodium sustained-release, Pharmaceutical quality, Saudi market, Dissolution profile, Generic brands

## Abstract

**Objective:**

We previously reported the pharmaceutical quality of eight brands of 50 mg enteric-coated diclofenac sodium tablet available on the Saudi market. Here, we assess the quality of reference (R1) and four generic (G1–G4) brands of 50 mg immediate-release diclofenac potassium tablet and of reference (R2) and generic (G5) brands of 100 mg sustained-release diclofenac sodium tablet.

**Results:**

Weight variation (range as % difference from mean), active substance content (mean (SD) as % difference from label), breaking force [mean (SD)], and friability (as % weight loss) were 95–104% and 99–102%, 100.9% (3.4%) and 105.6 (4.2%), 12.2 (1.3) and 12.9 (1.8) kg, and 0.0014% and 0.0012%, for R1 and R2, respectively. For G1-G5, they were ≤ ± 2%, 98.8% (2.7%) to 109.2% (3.8%), 6.4 (0.6) to 13.3 (1.0) kg, and 0.0007% to 0.0261%, respectively. R1 and G1-G4 disintegrated within 04:50–17:20 min: seconds and released a mean of 89–100% of label active substance content by 60 min in buffer (pH 6.8). R2 and G5 did not disintegrate or dissolve in 0.1 N HCl for 2 h, disintegrated in buffer (pH 6.8) in 01:58–02:15 h: minutes, and fulfilled dissolution criteria (pH 7.5) for both United States Pharmacopoeia test-1 and test-2. Thus all seven brands met pre-specified quality criteria.

## Introduction

Although the availability of generic drug products has been shown to expand healthcare accessibility and delivery [1], their quality is not infrequently questioned [2, 3].

To gain marketing approval in Saudi Arabia, generic drug products must pass standard bioequivalence testing [4]. Nevertheless, ongoing evaluation of marketed products remains crucial to protect public health and retain public and clinicians’ confidence. Such evaluation can be accomplished by in-vivo bioequivalence studies [5] or by in-vitro testing. In-vitro testing saves money and time, avoids involvement of research subjects, can forecast in-vivo bioavailability, and can substitute for bioequivalence studies of certain products [6–9].

Diclofenac is a non-steroidal anti-inflammatory drug [10] that is widely marketed in Saudi Arabia [4, 11]. It is commercially available for oral administration as sodium or potassium salts, and as immediate-release, enteric-coated, and sustained-release tablet formulations. Although some of the in-vitro quality standards, such as for weight variation, friability, and active substance content (ASC) tests are active substance- and formulation-independent, the standards for the most important tests, disintegration and dissolution tests, are active substance- and formulation-specific, both in testing conditions and acceptance criteria.

We previously reported the pharmaceutical quality of 50 mg enteric-coated diclofenac sodium tablet formulations available on the Saudi market [12]. Here, we assess the quality of the other diclofenac tablet formulations on the Saudi market, namely, 50 mg immediate-release diclofenac potassium tablet and 100 mg sustained-release diclofenac sodium tablet.

## Drugs and chemicals

We assessed all single-drug brands of 50 mg immediate-release diclofenac potassium tablet (R1 and G1–G4) and of 100 mg diclofenac sodium sustained-release tablet (R2 and G5) that were commercially available in Riyadh, Saudi Arabia between January 2020 and August 2020. Label information of the studied brands is presented in Table 1S (Additional file 1, Label information). There were three additional 50 mg immediate-release diclofenac potassium generic brands and one additional 100 mg diclofenac sodium sustained-release generic brand on the Saudi Formulary [4] that were not commercially available at the time of the study.

We purchased diclofenac standard from Sigma-Aldrich, St Louis, MO, USA; HPLC grade methanol and acetonitrile from Fisher Scientific Co., Loughborough, UK; disodium hydrogen phosphate from Fluka, Buchs, Switzerland; glacial acetic acid and potassium phosphate monobasic from Fisher Chemical, Fair Lawn, New Jersey, USA; and hydrochloric acid (HCl) from Merck, Darmstadt, F.R. Germany.

### Instruments

The instruments used in this study included High Performance Liquid Chromatography (HPLC)-dissolution system consisting of Waters 2690D Separation Module, Hanson Research SR8-Plus, United States Pharmacopoeia (USP) dissolution apparatus II (paddle), and Waters 996 Photodiode array detector set at 276 nm from Waters Associates Inc., Milford, MA. USA; Mettler AT20 sensitive balance from Mettler Toledo, Greifensee, Switzerland; and Model SSE-731Microprocessor Disintegration Test Apparatus, Model SSE-710 Microprocessor Friability Apparatus, and Model SSE-DIGIT AB-SPV Digital Tablet Hardness Tester from Sunshine Scientific Equipment, Delhi, India.

### Sample preparation and HPLC assay

We prepared a 1000 µg/ml stock solution of diclofenac sodium in methanol and diluted it in a phosphate buffer (pH 6.8 ± 0.05) composed of 0.05 M disodium hydrogen phosphate and 0.05 M potassium dihydrogen phosphate (50:50, v:v) to produce standard curve samples (0.1, 0.5, 1.0, 5.0, 10.0, 20.0, 40.0, 60.0 and 80.0 µg/ml for analyzing 50 mg tablets and 5, 10, 20, 40, 60, 80, 100, and 130 µg/ml for analyzing 100 mg tablets) and quality control samples (1.5, 7.5, 15, and 50 μg/ml for analyzing 50 mg tablets and 7.5, 15, 50, 70, and 115 μg/ml for analyzing 100 mg tablets). A previously reported HPLC assay [13] was used to determine ASC and dissolution profiles. The assay uses Nova-Pak C18 4-µm cartridge and a mobile phase composed of 0.2% glacial acetic acid (pH 3.0) and acetonitrile (51:49, volume: volume). There was no interference from tablet’s excipients. We used a standard curve and three sets of quality control samples in each run.

The above phosphate buffer (pH 6.8) was also used in disintegration testing and in dissolution testing of R1 and G1–G4. A 0.05 M phosphate buffer (pH 7.5 ± 0.05) was used in dissolution testing of R2 and G5.

### Quality control tests and calculations

For weight variation, friability, and ASC tests 20 randomly-selected units of each brand were examined. For the weight variation test, we calculated mean (SD) and % deviation of individual unit weight from mean weight of the brand. For the friability test, units were weighted, placed in a friabilator operated at 25 revolutions/minute for 4 min, then weighted again after de-dusting. We determined friability as % weight loss. For the ASC test, the units were individually crushed, dissolved in 100 ml methanol, filtered with a syringe using 0.2 µm filter, diluted with phosphate buffer (pH 6.8), and 100 µl were injected into the HPLC system. We calculated mean (SD) content and percent deviation of individual units from label.

For tablet breaking force test, 10 randomly-selected units of each brand were examined and mean (SD) pressure required to break each unit was determined.

Six randomly-selected units of each brand underwent disintegration testing. Phosphate buffer (pH 6.8) was used as disintegration medium for R1 and G1–G4 and 0.1 N HCL for 2 h followed by phosphate buffer (pH 6.8) for R2 and G5. We placed the basket rack in a 1000 ml vessel containing 900 ml of the disintegration medium (37 ± 2 °C). The basket rack moved 5–6 cm up and down (31 cycles/min) with the test unit remaining 1.5 cm below liquid surface and 2.5 cm above beaker bottom. We determined range of disintegration time (time to no particle on the basket).

Eight randomly-selected units of each brand initially underwent dissolution testing. If one or more units failed, additional 4 units were examined. For R1 and G1–G4, 900 ml phosphate buffer (pH 6.8) was used as dissolution medium. For R2 and G5, 900 ml 0.1 N HCL for 2 h followed by phosphate buffer (pH 7.5) were used as dissolution media. Stirring rate was 50 ± 1 rounds per minute (rpm) and temperature was set at 37 ± 0.5 ºC. The test ended with a stirring rate of 250 rpm for 15 min for R1 and G1-G4 and for one hour for R2 and G5. One ml sample was withdrawn midway between dissolution medium surface and rotating blade top, ≥ 1 cm away from vessel wall, and was immediately replaced with fresh medium. The samples were withdrawn at 5, 10, 15, 20, 30, 45, 60, 90 and 105 min in phosphate buffer for R1 and G1–G4, and at 60 and 120 min in 0.1 N HCl and 1, 2, 4, 5, 6, 10, 16, 24, and 25 h in phosphate buffer for R2 and G5. We kept the vessels covered, verified mixture temperature, and observed unit’s behavior throughout the test. We injected 100 µl of the one ml samples into the HPLC system. We determined mean (SD) amount released and % of label ASC released, at each time point.

## Results

Table 1 summarizes the main results. Mean weight of the seven brands ranged from 204.8 (1.7) to 317.0 (8.1) mg. Weight range was 95–104% of mean weight for R1, 99–102% for R2, and within ≤ ± 2% of mean weight for G1–G5. Mean (SD) ASC for R1 and R2 was 100.9% (3.4%) and 105.6% (4.2%) of label, respectively, and ranged from 98.8% (2.7%) to 109.2% (3.8%) for G1-G5.

**Table 1:** In-vitro quality of two reference and five generic diclofenac tablet brands available on the Saudi market

Code	Weight, n = 20		Active substance content^b^, n = 20		Breaking force, n = 10	Friability^c^, n = 20	Disintegration^d^ Phosphate buffer, n = 6	Dissolution^e^ Phosphate buffer, n = 8
	Mean (SD), mg	Range^a^, % from mean	Mean (SD), mg	Mean (SD), % of label	Mean (SD), kg/cm^2^	% loss	Range Hour: minute: second	Mean (range) release at 60 min, % of label
R1	317.0 (8.1)	95–104	50.5 (1.7)	100.9 (3.4)	12.2 (1.3)	0.0014	00:13:25 to 00:14:30	94 (89–99)
G1	262.4 (2.1)	99–102	54.6 (1.9)	109.2 (3.8)	9.3 (0.4)	0.0013	00:14:30 to 00:17:20	96 (84–104)
G2	264.6 (2.6)	99–102	51.1 (2.8)	102.1 (5.7)	6.4 (0.6)	0.0009	00:07:05 to 00:08:55	95 (66–103)^g^
G3	204.8 (1.7)	98–102	49.4 (1.3)	98.8 (2.7)	13.3 (1.0)	0.0012	00:05:10 to 00:05:50	89 (78–94)^g^
G4	205.6 (1.6)	99–101	50.2 (2.1)	100.4 (4.3)	11.4 (0.9)	0.0007	00:04:05 to 00:04:50	100 (93–105)
R2	302.3 (2.2)	99–102	105.6 (4.2)	105.6 (4.2)	12.9 (1.8)	0.0012	01:59:16 to 02:14:49^f^	See Fig. 1
G5	263.9 (2.9)	98–102	100.4 (3.9)	100.4 (3.9)	8.6 (0.5)	0.0261	01:58:02 to 02:13:56^f^	See Fig. 1

^a^Acceptable variation limits ≤ ± 7.5% for tablets > 80 and < 250 mg and ≤ ± 5% for tablets ≥ 250 mg; to pass, no more than 2/20 tablet differ by more than the percentage permitted and no one tablet differ by more than double the percentage

^b^Acceptable limits, mean content 90–110% of label

^c^Acceptable limit ≤ 1%

^d^pH 6.8

^e^For R1 and G1-G4, pH 6.8, acceptable limits, ≥ 75 + 5% of label. For R2 and G5, pH 7.5

^f^No disintegration was observed in 0.1 N HCL for 2 h

^g^Mean (range) of 12 rather than 8 units

Mean (SD) breaking force was 12.2 (1.3) kg for R1 and 12.9 (1.8) kg for R2 and ranged from 6.4 (0.6) to 13.3 (1.0) kg for G1–G5. R1 and R2, respectively, lost 0.0014% and 0.0012% of their weight during friability testing. Friability weight loss of G1–G5 ranged from 0.007 to 0.0261%.

Since R1 and G1–G4 were immediate-release tablets, their disintegration and dissolution were not examined in 0.1 N HCL. They disintegrated in phosphate buffer (pH 6.8) within 17:20 min: seconds. The sustained-release tablets, R2 and G5, did not disintegrate in 0.1 N HCl for 2 h but disintegrated in phosphate buffer (pH 6.8) within 02:14:49 hours: minutes: seconds.

**Fig. 1:**
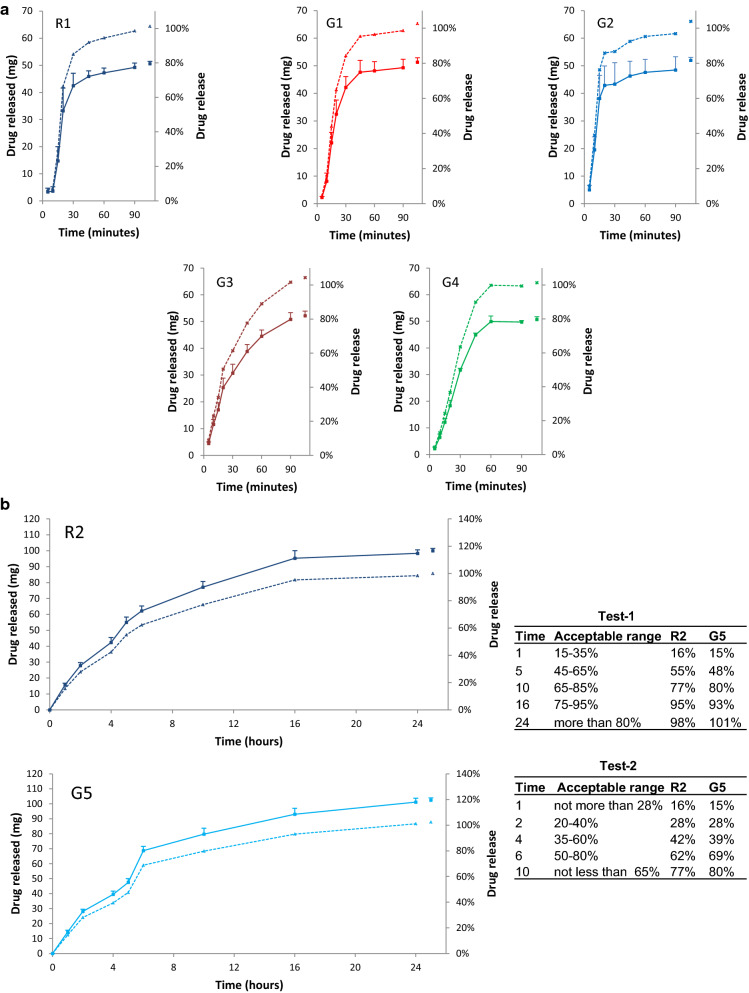
Dissolution profiles of two references and five generic diclofenac tablet brands available on the Saudi market. Mean (SD) amount of drug released at the specified times are shown on the left axis (continuous line) and percent of label amount released on the right axis (interrupted line). United States Pharmacopoeia (USP) dissolution apparatus type II (paddle apparatus) was used with a stirring rate of 50 ± 1 rpm (except for “infinity” time) and a temperature of 37 ± 0.5 ºC. Brands’ label details are available in Additional file 1, Label information. **a** 50 mg immediate-release diclofenac potassium tablet brands. R1, reference brand; G1–G4, generic brands. Dissolution medium was phosphate buffer (pH 6.8). Time 105 min indicates amount released with a stirring rate of 250 rpm for 15 min (“infinity”). **b** 100 mg sustained-release tablet brands. R2, reference brand; G5, generic brand. The insert shows the acceptable range of amount released at specified times according to USP test-1 and test-2 together with the amount released by R2 and G5. Time 0 min indicates amount released after 120 min in 0.1 N HCl. Other times indicate amount released in phosphate buffer (pH 7.5). Time 25 h indicates amount released with a stirring rate of 250 rpm for 1 h (“infinity”)

Dissolution profiles of R1 and G1–G4 are shown in Fig. 1a. Each of the 8 units of R1, G1, and G4 released ≥ 80% (Q (75%) + 5%) of label ASC within 60 min in phosphate buffer (pH 6.8) and thus met the acceptance criteria of S1 level [14]. However, because 1 of the 8 units of G2 and G3 released < 80% of label ASC by 60 min (66% and 78%, respectively), an additional 4 units were tested for each brand. Mean percent release for 12 units of G2 and G3 was 95% and 89%, respectively, meeting the acceptance criteria of S2 level [14].

Dissolution profiles for R2 and G5 are shown in Fig. 1b. There was 0.00% release in 0.1 N HCL for 2 h. In phosphate buffer (pH 7.5) timed percent release of each formulation fulfilled criteria for both test-1 and test-2 of USP [15]. We did not detect any artifacts such as floating material, coning, gumming, odd erosion pattern, air bubbles, or adhering particles during dissolution testing of R1, R2, or G1-G5.

## Discussion

We assessed the pharmaceutical quality of a reference and four generic 50 mg immediate-release diclofenac potassium tablet brands and a reference and a generic 100 mg sustained-release diclofenac sodium tablet brands that were commercially available on the Saudi market. All brands passed in-vitro quality testing according to USP [15]. Namely, weight variation of ≤ ± 5% from mean weight; mean ASC between 90 and 110% of label; ≤ 1% friability weight loss; complete disintegration in phosphate buffer (pH 6.8) within 60 min for the immediate-release brands and no disintegration in 0.1 N HCl for 2 h for the sustained-release brands; and release of ≥ 80% within 60 min in phosphate buffer (pH 6.5) for the immediate-release brands and timed release in phosphate buffer (pH 7.5) for the sustained-release brands.

The results of this study are consistent with previous results showing an acceptable quality of commercially available diclofenac tablet brands. We have previously evaluated in-vitro quality of a reference and 7 generic brands of 50 mg diclofenac sodium enteric-coated tablet that were commercially available on the Saudi market, except for borderline performance of one generic brand, all brands passed in-vitro quality testing according to USP [12]. Further, two studies each comparing dissolution profiles of a reference and five generic immediate-release diclofenac potassium 50 mg tablet brands marketed in Pakistan [16] and Argentina [17] found that they all fulfilled USP specifications. Furthermore, a study that examined 3 generic brands of 100 mg diclofenac sodium sustained-release tablet marketed in India found that all brands comply with established limits [18].

The current results together with the results of several pre-marketing [19–26] and post-marketing [5] in-vivo bioequivalence studies on other drug products provide assurance of the quality of generic drug products marketed in Saudi Arabia.

## Study strengths

The study assessed all available brands, included two reference brands, examined multiple-point dissolution curves, and used a validated HPLC assay (rather than just a spectrophotometer) to determine diclofenac level, with the advantage of avoiding interferences from formulation matrix/dissolution medium and detecting potential drug degradation.

## Study limitations

Our results do not necessarily apply to all diclofenac tablet brands on the Saudi formulary. Three immediate-release diclofenac potassium tablet brands and one sustained-release diclofenac sodium tablet brand were listed on the Saudi Formulary but were not included in the current study because they were not available in Riyadh pharmacies at the time of the study. Further, our results do not apply to diclofenac formulations of other strength or form.

## Supplementary information

**Additional file 1: Table 1S.** Label information of two reference and five generic diclofenac tablet brands available on the Saudi market.

## Data Availability

Additional data are available in Table 1S (Additional file 1, Label information). Raw data are available from the corresponding author upon request.

## Abbreviations

ASC: Active substance content
G: Generic brand
HCL: Hydrochloric acid
HPLC: High performance liquid chromatography
R: Reference brand
rpm: Rounds per minute
SD: Standard deviation
USP: United States Pharmacopoeia
