# Supplementary material for: Pharmaceutical quality of seven brands of diclofenac tablet on the Saudi market
Source: BMC Res Notes. 2020 Nov 26;13:548. doi: 10.1186/s13104-020-05385-8 (PMC7694918; doi:10.1186/s13104-020-05385-8)
Supplement: Supplementary file 1 — Additional file 1: Table 1S. Label information of two reference and five generic diclofenac tablet brands available on the Saudi market. [file 13104_2020_5385_MOESM1_ESM.docx]

| **Code** | **Manufacturer** | **Manufacture date** | **Expiration date** | **Batch /lot number** | **Date Assessed** | **Active substance** | **Trade name** | **Form** | **Dose**  **(mg)** |
| --- | --- | --- | --- | --- | --- | --- | --- | --- | --- |
| R1 | Novartis | 4/2019 | 4/2021 | KM872/B | 7/2020 | Diclofenac potassium | Cataflam | Tablet | 50 |
| G1 | Pharma International | 2/2019 | 2/2022 | 1997 | 7/2020 | Diclofenac potassium | Dolvic-K | Tablet | 50 |
| G2 | Tabuk Pharmaceutical Mfg. Co. | 3/2020 | 3/2023 | OMX612 | 7/2020 | Diclofenac potassium | Rapidus | Film coated tablet | 50 |
| G3 | Oman Pharmaceutical Products CO. L.L.C. | 12/2019 | 12/2021 | 9CH024A | 8/2020 | Diclofenac potassium | CLOFAST-P | Film coated tablet | 50 |
| G4 | Jamjoom Pharma | 9/2019 | 9/2021 | WK0082 | 8/2020 | Diclofenac potassium | Fast-Flam | Film coated tablet | 50 |
| R2 | Novartis | 4/2019 | 4/2022 | TAJ19 | 7/2020 | Diclofenac sodium | Voltaren Retard | Sustained-release tablet | 100 |
| G5 | SPIMACO | 9/2019 | 9/2022 | 118163 | 7/2020 | Diclofenac sodium | ROFENAC S.R. | Sustained-release tablet | 100 |

**Table 1S:** Label information of two reference and five generic diclofenac tablet brands available on the Saudi market
